# Barriers and facilitators to the implementation of workplace health promotion programs: Employers' perceptions

**DOI:** 10.3389/fpubh.2022.1035064

**Published:** 2023-01-12

**Authors:** Jennifer M. D. Campmans, Denise J. M. Smit, Sandra H. van Oostrom, Josephine A. Engels, Karin I. Proper

**Affiliations:** ^1^Center for Nutrition, Prevention and Health Services, National Institute for Public Health and the Environment, Bilthoven, Netherlands; ^2^Department of Public and Occupational Health, Amsterdam UMC, Vrije Universiteit Amsterdam, Amsterdam Public Health Research Institute, Amsterdam, Netherlands; ^3^Occupation & Health Research Group, HAN University of Applied Sciences, Nijmegen, Netherlands

**Keywords:** workplace health promotion programs, employers' perspectives, implementation, consolidated framework for implementation research, qualitative study, integrated approach

## Abstract

**Background:**

Workplace health promotion programs (WHPPs) can benefit the lifestyle and health of employees. However, not all WHPPs have been successful in their implementation, and thus their effectiveness. This study aimed to identify the barriers and facilitators to implementing an integrated WHPP, which targets multiple lifestyle factors at different levels (individual and organizational), from an employer's perspective.

**Methods:**

Data were collected by two online focus groups among 18 representatives of eight different organizations. Data from the focus group discussions were transcribed verbatim and analyzed using thematic analysis. Data were coded both inductively and deductively, using the Consolidated Framework for Implementation Research (CFIR) consisting of the following five domains: (1) intervention characteristics, (2) outer setting, (3) inner setting, (4) characteristics of individuals, and (5) process. Ratings were performed to indicate the positive or negative influence and strength of a construct regarding the implementation of WHPPs.

**Results:**

Barriers and facilitators in all domains of the CFIR were found. Regarding characteristics of the WHPP, complexity and costs hindered implementation, while high adaptability facilitated it. An organization that met the needs of employees (the outer setting) facilitated implementation. Available resources, access to knowledge, leadership involvement, and continuity of communication were facilitators within the inner setting. Barriers were different approaches to implementation within one organization and the perceived interference with employees' lives. For the implementation process, the involvement of key stakeholders, including employees, was identified as an important facilitator.

**Conclusion:**

Various barriers and facilitators in different domains play a role in the implementation of integrated WHPPs, according to employers. Strategies that tackle the identified barriers and incorporate the facilitators will likely contribute to the successful implementation of integrated WHPPs.

## 1. Introduction

The workplace is described as a promising environment to encourage people to make healthier lifestyle choices (1, 2). Workplace health promotion programs (WHPPs) aim to improve lifestyle and consequently health- and work-related outcomes (3). The effectiveness of these programs has been investigated in numerous studies (4, 5). For example, Verweij et al. (4) found significant effects on the reduction of body weight, BMI, and body fat Moreover, increased productivity rates, decreased absenteeism, and therefore a reduction in associated costs are potential benefits for employers (6).

Despite proven positive effects, not all WHPPs that have been implemented over the years have been successful (7). The difficulty of the translation from research to practice has been acknowledged (8). Health promotion interventions that are based on proper underlying theory may not yield positive effects in practice as a consequence of poor implementation (8).

For example, inadequate implementation strategies may contribute to poor compliance and low participation rates of employees and hence a lack of effectiveness (9). Participation rates in WHPPs differ across studies from 8 to 97% but are on average low, with participation levels below 50% (10, 11). To maximize participation levels and thereby increase the success rate of a WHPP, the implementation process should be carefully considered, as was emphasized in earlier research (9, 11, 12). To date, no firm conclusions about strategies to improve the implementation of WHPPs can be drawn, as implementation research on WHPPs is “only just emerging” and more research is warranted (13).

To achieve successful implementation, a needs assessment, including an assessment of barriers and facilitators regarding the implementation of a WHPP, is needed (5). As employers are key stakeholders in this, it is critical to consider their perspective on the factors that impede or facilitate implementation (5, 14).

This study was conducted in preparation for the development, implementation, and evaluation of an integrated WHPP in which a European good practice, the “Lombardy Workplace Health Promotion (WHP) Network,” was tailored to the Dutch context (15). The success factor of the Lombardy program was the integrated approach to promote multiple healthy behaviors at both the individual and organizational levels (16). This study aimed to identify barriers and facilitators to the implementation of a Dutch-integrated WHPP by assessing the experiences of employers with implementing WHPPs.

## 2. Material and methods

### 2.1. Study design

A qualitative design consisting of focus groups with representatives of employers was used. In focus groups, interaction and discussion between group members are stimulated, which leads to extra information and collective views on a topic (17).

### 2.2. Study population

The study population consisted of 18 employers or representatives from eight Dutch organizations who had experience in implementing a WHPP. Job positions included director, human resources (HR) officer, and manager. All organizations had more than 250 employees. Based on the International Standard Industrial Classification of All Economic Activities (ISIC) (18), the participating employers were from different sectors, as presented in Table 1. In total, 10 employers participated in the first focus group and eight employers in the second.

**Table 1:** Characteristics of participants (*n* = 18).

	**NO. (%)**
**Industry**	
Administrative and support service activities	2 (11.1%)
Electricity, gas, steam and air conditioning supply	2 (11.1%)
Accommodation and foodservice activities	3 (16.7%)
Public administration and defense; compulsory social security	3 (16.7%)
Manufacturing	3 (16.7%)
Education	5 (27.8%)
**Job title**	
Advisor	3 (16.7%)
Director	1 (5.6%)
HR officer	2 (11.1%)
Manager	9 (50%)
Policy officer	2 (11.1%)
Prevention officer	1 (5.6%)

Organizations were recruited *via* the networks of the project team members, co-workers, and branch-specific networks and platforms. At first, announcements to participate in an intervention study in which an integrated WHPP would be implemented were distributed through online platforms. A total of 13 organizations were interested and responded by e-mail; out of those, nine organizations were approached for this study, and eight of them decided to participate. For the recruitment of organizations, purposive sampling was used to pursue different organizations with blue-collar and/or white-collar employees (19). Additionally, representatives from the organizations were recruited to participate in the focus group through snowball sampling within the organization (19). An e-mail with practical information was sent to the organizations that participated in the focus groups.

### 2.3. Data collection

A semi-structured interview guide was developed and aimed to identify the barriers and facilitators to the implementation of WHPPs based on employers' experiences. The topics in the interview guide included (1) determinants that facilitated the implementation and (2) determinants that hindered the implementation of a WHPP. Questions such as “What factors caused the implementation to be effective?” were asked. Because of the COVID-19 pandemic restrictions, the focus groups were conducted online using “GoToMeeting” and “Microsoft Teams.” Both focus groups had a duration of 90 min and were recorded with the permission of the respondents. Oral consent was obtained from all participants. The focus groups were conducted by one facilitator (DS). Two assistants took notes and managed time (JC and SO). To facilitate the active participation of all employers, they were asked to write down barriers and facilitators to implementation individually during the focus group. Every participant then reported one factor, which was noted down using an online whiteboard. These factors were then discussed, and missing factors were added.

### 2.4. The theoretical framework for qualitative analysis

The Consolidated Framework for Implementation Research (CFIR) was used to identify the relevant factors for the implementation of a WHPP in the pre-implementation phase (21). The CFIR is composed of the following five domains: (1) intervention characteristics, (2) outer setting, (3) inner setting, (4) characteristics of individuals, and (5) process. “Intervention characteristics” involved the features of the WHPP itself, the “outer setting” of the external environment (22). “inner setting” concerned features of the implementing organization (22). The fourth domain was used to explore “individual characteristics” of the implementers that might influence implementation, and “process” contained the strategies involved in the implementation (22).

### 2.5. Data analysis

The data from the focus group discussions were transcribed verbatim and analyzed with MAXQDA 2020. A thematic analysis was used, as described by Braun and Clarke (23). Moreover, a hybrid process of deductive and inductive coding was used for the analysis (24). The data were analyzed by two researchers (JC and DS) separately, then compared and discussed, and in case of disagreement, discussed with a third researcher (SO) to reach a consensus. The first step was to familiarize ourselves with the data by reading the transcripts of the focus groups. In step two, an initial codebook was formed deductively based on the CFIR's five domains and constructs. The combination with the inductive approach offered the possibility of including new codes that emerged from the data (24). The third step was the organization of themes and codes, wherein sections of the data that represented the same code were gathered. In the fourth step, all themes and codes were reviewed and reconsidered. In the fifth step, the themes were further refined, and the essence of each theme and construct was clarified. Finally, the CFIR's constructs were ranked independently by two researchers (JC and DS) (20). The ratings reflect the valence, implying whether the construct hampered or facilitated implementation, and the strength, ranging from −2 to +2 (20). The rating criteria used for this study are shown in Table 2. The data analysis was an iterative process, as the first focus group was analyzed before the second focus group was conducted (25).

**Table 2:** Criteria used to assign ratings to the constructs, based on the CFIR framework (20).

**−2**	**The construct is a negative influence in the organization, an impeding influence in work processes, and/or an impeding influence in implementation efforts. The majority of employers describe explicit examples of how the key or all aspects (or the absence) of a construct manifests itself in a negative way**
−1	The construct is a negative influence in the organization, an impeding influence in work processes, and/or an impeding influence in implementation efforts. Employers make general statements about the construct manifesting in a negative way but without concrete examples: - The construct is mentioned only in passing or at a high level without examples or evidence of actual, concrete descriptions of how that construct manifests - There is a mixed effect of different aspects of the construct but with a general overall negative effect - There is sufficient information to make an indirect inference about the generally negative influence; and/or - Judged as weakly negative by the absence of the construct
X	The construct can have a mixed rating if: - The comments are equally positive and negative
+1	The construct is a positive influence in the organization, a facilitating influence in work processes, and/or a facilitating influence in implementation efforts. Employers make general statements about the construct manifesting in a positive way but without concrete examples: - The construct is mentioned only in passing or at a high level without examples or evidence of actual, concrete descriptions of how that construct manifests - There is a mixed effect of different aspects of the construct but with a general overall positive effect; and/or - There is sufficient information to make an indirect inference about the generally positive influence
+2	The construct is a positive influence in the organization, a facilitating influence in work processes, and/or a facilitating influence in implementation efforts. The majority of employers describe explicit examples of how the key or all aspects of a construct manifests itself in a positive way

## 3. Results

**Figure 1:**
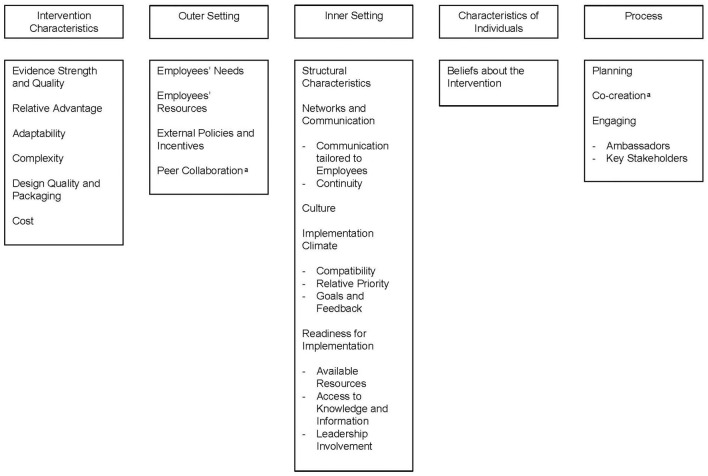
Overview of the condtructs in which barriers and facilitators were identified, categorized according to the five domains of the CFIR. ^a^Emerged inductively as a construct.

In total, barriers and facilitators were identified in 25 constructs (Figure 1). The ratings of the constructs are presented in Table 3.

**Table 3:** Barriers and facilitators to the implementation of a WHPP.

**Domain**	**Construct**	**Rating**
Intervention characteristics	Evidence Strength and Quality	+2
	Relative Advantage	+1
	Adaptability	+2
	Complexity	−2
	Design Quality and Packaging	+1
	Cost	−1
Outer setting	Employees' Needs	+1
	Employees' Resources	X
	External Policies and Incentives	+1
	Peer Collaboration^a^	+1
Inner setting	Structural Characteristics	−2
	Networks and Communications
	- Communication tailored to employees	X
	- Continuity	+2
	Culture	−2
	Implementation Climate
	- Compatibility	+1
	- Relative Priority	+2
	- Goals and Feedback	X
	Readiness for Implementation
	- Available Resources	+2
	- Access to Knowledge and Information	+2
	- Leadership Involvement	+2
Characteristics of individuals	Beliefs about the Intervention	−1
Process	Planning	+2
	Co-creation^a^	+2
	Engaging
	- Ambassadors	+1
	- Key stakeholders	+1

^a^Emerged inductively as a construct.

### 3.1. Intervention characteristics

#### 3.1.1. Facilitators

Constructs related to the characteristics of the program that had a strong positive influence (+2) on implementation according to multiple employers were **Evidence Strength and Quality** and **Adaptability**.

Employers preferred a WHPP that was evidence-based, as they believed this increased the chance of successful implementation. Moreover, evidence of the usefulness of the WHPP was helpful in engaging other stakeholders:

“*We are responsible for convincing people about the usefulness of the program. A program must have a guide for the promotion and a description of the usefulness and necessity, as this helps people to sell it [the program] in the organization.”—[Manufacturing, manager]*

It was indicated that it should be possible that a program can be adapted to meet the needs of the target group, in this case, employees. Examples of the adaptability of a WHPP included programs that were suitable to the employees' life stages. For instance, a program that incorporates themes based upon the employee's life stage, such as work-life balance for younger employees and sustainable employability for older employees, has high adaptability and therefore meets the local needs of the employees. Other WHPPs that were adaptive and thus facilitated implementation included WHPPs that were intertwined with the employee's job, e.g., by adapting the content to the nature of their work.

The constructs **Relative Advantage** and **Design Quality and Packaging** were assigned +1.

It was indicated that if the impact of WHPPs within the organization was assessed beforehand, this was helpful in prioritizing which WHPPs to implement. To improve the design and quality of the WHPP, it was mentioned that the use and integration of professionals' knowledge into the design served as a facilitator:

“*We have used the knowledge of physiotherapists and physical therapy students in our project to give substance to our intervention. We know several things, but these professionals can convey it in a better way, in a clearer way, and they will also be received differently […] by the employees.”—[Administrative and support service activities, policy officer]*

Employers found the enrollment of employees to be both a facilitator and a barrier. It was seen as a facilitator if the enrollment was made accessible and easily done, for instance by automatic enrollment in the WHPP instead of employees having to sign up themselves. Enrollment through the supervisor or other individuals in leadership roles was experienced as a threshold for employees. Furthermore, there were different opinions regarding the obligatory nature of WHPPs. One employer emphasized the importance of voluntariness, as not all employees are into lifestyle changes or do not realize they need to improve them, while the latter was a reason for another employer to make WHPPs obligatory.

#### 3.1.2. Barriers

The constructs that negatively influenced the implementation were **Complexity** (−2) and **Cost** (−1). The majority of employers expressed the complexity of a WHPP to be implemented as a barrier. WHPPs that had simple, practical approaches to implementation were considered facilitating:

“*Success factors that I have experienced are sufficient and continuous attention for guidance and coaching and for the supervisors especially a concise and practical approach.”—[Education, manager]*

One employer mentioned costs related to the implementation of the WHPP as a barrier.

### 3.2. Outer setting

#### 3.2.1. Facilitators

The constructs **Employees' Needs, External Policies and Incentives**, and **Peer Collaboration** had an overall positive influence on implementation and were ranked +1.

The construct employees' needs concerned the extent to which employees' needs are accurately known and met by the organization (22). Employers acknowledged that WHPPs that meet the needs of the target group, serve as a facilitator. An example was:

“*Once, the organization came up with the idea of making a healthy canteen overnight. […] Consequently, all employees went to the local snack bar. This was not very effective, and eventually, the menu was adjusted, so the meatballs returned. […] So, the target group really must want it. […]”—[Accommodation and foodservice activities, HR officer]*

As for external policies, an employer stated that it was easy to impose a government policy regarding smoking cessation on employees. Another employer mentioned using a legal obligation in WHPPs as a facilitator. Furthermore, peer collaboration referred to the created learning situation when other organizations have already implemented WHPPs and exchanged their experiences to help other organizations implement WHPPs.

#### 3.2.1. Mixed constructs

**Employees' Resources** (X) were addressed as a barrier and facilitator. The provision of an activity outside the organization was mentioned as a threshold to attend the activity. Moreover, a lack of time for employees and extra costs to be paid by employees were barriers, e.g., if healthy food offered in the canteen was more expensive than unhealthy food. On the contrary, when the organization prioritized the employees' resources and thus provided healthy foods for a reduced price, a favorable behavior change was seen.

### 3.3. Inner setting

#### 3.3.1. Facilitators

Readiness for implementation involved **Available Resources**, **Access to Knowledge and Information**, and **Leadership Involvement**. These were all reported as important facilitators (+2).

Multiple available resources, such as the need to provide WHPPs during working time, locations specifically designated for the WHPP, and a budget, were facilitators. Moreover, a success factor was to inform and educate supervisors separately on how to promote a healthy lifestyle for their employees. Examples given were a training or implementation guide for supervisors to support them in the implementation:

“*It would be nice, if there was a manual or something for supervisors, with information about how you make these kinds of topics discussable. […] About how you stimulate employees to take that break or adopt a healthier lifestyle. These are often difficult things to discuss because it is often what you interfere with. So, I notice that supervisors need help with that.”—[Education, policy officer]*

Leadership involvement, the involvement of leaders and managers with the implementation, was found to be an important aspect in the implementation of WHPPs:

*[…] I think that supervisors are very important. What we notice is that people give up quickly when they're busy. They say: ‘well, I don't have time for this.' But if a supervisor encourages them […], people are more inclined to do it.”—[Public administration and defense; compulsory social security, manager]*

Additionally, supervisors who were actively engaged in the project and supported the employees during the participation in WHPPs facilitated participation.

**Continuity**, part of the construct Networks and Communications, emerged as a strong facilitator (+2).

The power of repetition was mentioned by multiple employers. Involvement and motivation rose when information was provided continuously:

“*And so that it's also a permanent theme for them, permanently under the attention. You must repeat things more structurally or be present in a structured way to bring that theme to their attention continuously.”—[Administrative and support service activities, policy officer]*

**Compatibility** (+1) and **Relative Priority** (+2), both part of the construct Implementation Climate, were ranked as facilitators. One employer mentioned that a WHPP that was compatible with and fitted with existing workflows and policies was considered a facilitator. Priority given by stakeholders, such as top management, facilitated getting the implementation of WHPPs on the agenda.

#### 3.3.2. Barriers

**Structural Characteristics** and **Culture** were identified as barriers (−2) to the implementation, according to the majority of employers.

Scattered health promotion initiatives were mentioned as a barrier, as this caused uncertainty for employees about what was offered. Different approaches within the company hindered the implementation of a WHPP throughout the whole company:

“*A barrier is too many different policies. We have six clusters within [organization]. […] One has a vitality coordinator, the other has its working group, and it's quite difficult to find our way, as an organizational-wide program. So, I'm not sure if that's because of different policies, but it's maybe due to a lot of different approaches.”—[Public administration and defense; compulsory social security, manager]*

Culture referred to the perceived interference with the employees' private lives, as was expressed by an employer:

“*I think because that is seen as very patronizing as if you are interfering. I think that responsibility should also lie with the employees themselves. It will only work if an employee feels like it is important and is willing to work on it, because had I asked, ‘What do you do about your health?', then he [employee] says: ‘You know, I work from 9 to 5, you can interfere with that, but outside of that you can't'.”—[Education, manager]*

#### 3.3.3. Mixed constructs

The constructs **Communication Tailored to Employees**, part of Networks and Communications, and **Goals and Feedback** were experienced to both hinder and facilitate implementation (X).

It was mentioned that communication should be tailored to the characteristics of the target group, the employees. A barrier was the use of a single communication channel, whereas the use of multiple communication channels was considered a facilitator. Delivering information to employees came forward as a difficult aspect:

“*[...] My biggest frustration is that no matter what I do, I can't get it between the ears of the employees. With whatever campaign I'm running.”—[Education, prevention officer]*

To inform implementers of the program about the goals of the WHPP and to provide clarity regarding the expectations of a WHPP were facilitators. According to employers, goals were not always communicated clearly to implementers that was considered a barrier:

“*It is very important to transfer the information and its purpose very clearly from within our organization to the intermediary, who eventually has to transfer it to the target group. Because if something is missing or not indicated in a structured way or insufficiently, tight and clear, then it also does not come across well to the target group. We noticed that in our interventions, and then you still have to intervene as an organization.”*—[*Administrative and support service, policy officer]*

### 3.4. Characteristics of individuals

#### 3.4.1. Barriers

As to the construct **Beliefs about the Intervention** (−1), it was identified that a negative attitude of the supervisor toward WHPPs might hamper implementation even more than a positive attitude facilitating the implementation:

“*Here it's really on the supervisor and I think that this works even stronger than the positive side. So, if the supervisor has a negative attitude, it is difficult for the employees to ignore that and still go or work on it. [...] And if a supervisor has a positive attitude it's okay, also if the supervisor is neutral, but negative is a disaster.”—[Electricity, gas, steam and air conditioning supply, manager]*

One employer added that it worked adversely when supervisors do not feel the need for the program and felt like they got extra work. In contrast, those supervisors that are advocates of a WHPP and believe in the potential of a WHPPs could just improve employees' work functioning.

### 3.5. Process

#### 3.5.1. Facilitators

**Planning** and **Co-creation** strongly and positively influenced (+2) implementation. **Engaging Key Stakeholders** and **Engaging Ambassadors** had an overall positive influence on the implementation (+1).

As to the planning, it is important to be clear about the time path of the implementation and to start early with the involvement of stakeholders within the organization. The facilitating effect of a quick implementation after the development process was expressed:

“*Collecting information and turning it into action, we did that relatively quick. So, for the first six months, we collected information, and then for the second six months, we implemented it. We noticed that you should not wait too long with that. The implementation should follow quickly.”—[Administrative and support service activities, policy officer]*

Co-creation referred to the involvement of employees in the development and implementation of a WHPP so that the program better fits the needs of the target group. More specifically, ambassadors (enthusiastic employees) were mentioned to be involved. Co-creation facilitated implementation in all cases and was used by multiple employers:

“*We have collected and used input from the employees. Instead of imposing something top-down and thinking of it at a strategic level and then imposing it at an operational level. That often doesn*'*t work or creates bottlenecks.”—[Administrative and support service activities, policy officer]*

For an integrated WHPP especially, it was mentioned that it was important to involve key stakeholders. The following quote illustrates a lack of engagement of key stakeholders when there was little communication between the different departments:

“*We had it all set up, but at the factory, their line supervisor didn't give them the time off to participate in a workshop. […]. They also wanted to, but we forgot to coordinate with the factory itself that we would provide a workshop and time and space should be created for this, which is unnecessary for office workers for example.”—[Manufacturing, HR officer]*

## 4. Discussion

### 4.1. Main findings

In our study, the CFIR was used to identify barriers and facilitators to implementing an integrated WHPP. WHPPs that are evidence-based and have the potential to be tailored to the target group were facilitators, while complexity and costs were barriers. Within the organization, it appeared important to have available resources, access to knowledge and guidance, and leadership involvement. Barriers were different approaches to implementation and the perceived interference with employees' lives. As to the implementation process, having structured planning and co-creation facilitated implementation.

### 4.2. Comparison with literature

In line with our study, other studies that identified barriers and/or facilitators according to employers reported some similar findings (7, 26, 27). A lack of management support was a frequently mentioned barrier and a facilitator when support was present (7, 26, 27). The importance of supervisors' attitudes toward WHPPs and their involvement in their implementation also came up in our study. Furthermore, employers identified that a negative attitude can have a greater impact on implementation than a positive attitude as was confirmed in our study. A lack of knowledge about the importance of health promotion or seeing it as an extra workload can cause a supervisor's negative attitude toward the implementation of a WHPP (28). Therefore, programs that aim to improve knowledge and attitudes among supervisors regarding health promotion can benefit the implementation of WHPPs (29). These programs can provide guidance on how to organize WHPPs, for example, which was identified as a need in our study and the study of Ruiz-Dominguez et al. (30). While leadership involvement appeared facilitating, employers in our study mentioned that employees could perceive interference with their lives. Pescud et al. (31) mentioned that employers often do not feel the responsibility to improve their employees' health because they believe that employees should be responsible for their health. In our study, this was not identified, which could be a consequence of the fact that employers included were experienced with the implementation of WHPPs and thus had an affinity for health promotion among their employees.

Another facilitator identified for implementation, in line with our study, is good collaboration with all stakeholders involved (7). It is recommended to engage managers and employees (co-creation) early in the planning process to develop strategies to overcome implementation barriers (26, 30). Moreover, since employers reported that different approaches or policies regarding health promotion were a barrier to implementation, it can be concluded from our study that it is important to have a good overview of who is working on what within the organization regarding health promotion. “Employee” or “healthy workplace” committees have been proposed to enhance engagement. Different stakeholders can be represented, including employees from various departments in an organization, “ambassadors” who enthusiastically support WHPPs, and management (26, 27). Such committees can improve employee participation as their needs are known and met by the organization. Also, employees' resources should be considered in these committees, since these were not met in our study and led to implementation challenges. Additionally, committees can improve management support as managers are kept informed about the progress of the WHPP and show that they are committed to the success of the WHPP (26, 27).

Barriers and facilitators to the implementation of WHPPs, in line with our findings, were related to the organization's readiness and the availability of resources, respectively (7, 26, 27). Having a budget available was mentioned as a facilitator, which is in line with previous studies underlining the importance of resources at the organizational level, e.g., time, budget, and human resources (32, 33). In this study, costs were only mentioned by one participant as a barrier to implementation. Costs might be more of an issue in the decision-making about implementing WHPPs by the management of organizations. This might explain why costs are not often perceived as a barrier during the implementation phase. The extent to which resources are a challenge for the implementation of WHPPs depends on the type and size of the organization. Smaller organizations with fewer employees are less likely and less able than large organizations to offer WHPPs (34). However, WHPPs vary in terms of content and implementation costs. Smaller organizations can decide to start small with the implementation and scale up eventually (35).

### 4.3. Strengths and limitations

The main strength of this study is the identification of barriers and facilitators in the pre-implementation phase of an integrated WHPP by considering perceptions from employers about previously implemented WHPPs. In doing so, employers from different organizations with blue-collar and/or white-collar employees participated and represented a range of perspectives. Although representatives of employers were recruited based on previous experiences with the implementation of WHPPs to provide insights into barriers and facilitators, selection bias can be present as all participants feel the importance of health promotion in the workplace (36). Additionally, the organizations in this study had more than 250 employees, underrepresenting employers from small- and medium-sized enterprises (SMEs). Since SMEs have specific characteristics, such as time and resource constraints, other barriers and facilitators may play a role in the implementation of WHPPs in SMEs (37).

Due to the COVID-19 pandemic, both focus groups were online. The advantages of virtual focus groups are the relatively lower costs and the fact that participants can join in from their homes and do not need to travel (38). However, the interaction between participants might be different in online discussions. There may be either an increase in interaction due to the participants' feeling of anonymity or a decrease because of a potential loss of spontaneous reactions (39, 40). Moreover, researchers are limited in observing the participants' body language and receiving nonverbal signals (39, 41).

The hybrid process of deductive and inductive coding resulted in a rich number of constructs. The existing constructs of the CFIR provided a basis for identifying barriers and facilitators, but the new constructs “co-creation” and “peer collaboration” may be valuable additions to the CFIR within workplace settings (20, 42). Co-creation, also known as a “participatory approach” is also identified as a success factor for the implementation of WHPPs in other studies (43–45). In our opinion, the ratings are of added value, as they provide an overview of the valence and strength of the constructs. They are based on the input of the employers and reflect the frequency and consistency of themes raised during the focus groups. To avoid subjectivity in ratings, they were discussed extensively within the research team (46). Finally, as the domains of the CFIR are interrelated, it is difficult to make a clear distinction between the constructs as they can overlap. For example, both adaptability and co-creation aim to match the needs of the employees, but adaptability reflects a characteristic of the intervention and co-creation of the process in which the WHPP is developed and implemented.

### 4.4. Implications

The identified barriers and facilitators in all domains of the CFIR can help to reach effective implementation in future WHPPs. A WHPP that has high adaptability and matches the characteristics of the employees should be strived for. Moreover, since each work setting and employee population have their own inherent cultures and demands, thorough consideration should be given to these needs before implementing a WHPP. Therefore, it is crucial to involve management and employees in the implementation (7). Multiple channel communication and providing information continuously are key (29). This study builds upon the “Lombardy Workplace Health Promotion Network,” an integrated WHPP that addresses different lifestyle themes within various domains. From this study, we have learned to involve all stakeholders, e.g., the caterer for adjustments in the staff canteen, professionals for delivering knowledge about health behavior, and supervisors to support and motivate employees. Furthermore, as factors influenced implementation in all domains of the CFIR, this emphasizes the importance of using an integrated approach to implementation that focuses on all levels. Future research could also incorporate the views of employers who have less affinity with workplace health promotion.

## 5. Conclusion

In this study, various barriers and facilitators in different domains play a role in the implementation of WHPPs in the Dutch context according to employers were identified. Several strategies that tackle the identified barriers and incorporate the facilitators should be put into practice for the successful implementation of integrated WHPPs.

## Data availability statement

The datasets presented in this article are not readily available because the datasets generated and analyzed during the current study are potentially identifiable. These data are available from the corresponding author on reasonable request. Requests to access the datasets should be directed to denise.smit@rivm.nl. Information about participating organizations will not be provided.

## Ethics statement

The studies involving human participants were reviewed and approved by the Center for Clinical Expertise of the Dutch National Institute of Public Health and the Environment. Written informed consent was not provided because oral consent was obtained from all participants. This was video recorded prior to the focus groups.

## Author contributions

DS, SO, KP, and JE developed the interview guide. DS, SO, and KP collected the data of the first focus group. DS, SO, and JC collected data of the second focus group. JC and DS analyzed the data, after which SO was involved in the final decisions. JC wrote the initial manuscript. Findings were examined and discussed among all authors. All authors read and approved the final manuscript.

## References

[1] Kilpatrick M Blizzard L Sanderson K Teale B Jose K Venn A. Barriers and facilitators to participation in workplace health promotion (WHP) activities: results from a cross-sectional survey of public-sector employees in Tasmania, Australia. Health Promot J Austr. (2017) 28:225–32. 10.1071/HE16052 28110642

[2] Rongen A. Sustainable Employability & Participation in Health Promotion Programs. Rotterdam: Erasmus University Rotterdam (2015).

[3] Hendriksen I Snoijer M De Kok B Van Vilsteren J Hofstetter H. Effectiveness of a multilevel workplace health promotion program on vitality, health, and work-related outcomes. J Occup Environ Med. (2016) 58:575–83. 10.1097/JOM.0000000000000747 27136605 PMC4883645

[4] Verweij L Coffeng J van Mechelen W Proper K. Meta-analyses of workplace physical activity and dietary behaviour interventions on weight outcomes. Obes Rev. (2011) 12:406–29. 10.1111/j.1467-789X.2010.00765.x 20546142

[5] Proper K Van Oostrom S. The effectiveness of workplace health promotion interventions on physical and mental health outcomes - a systematic review of reviews. Scand J Work Environ Health. (2019) 45:546–59. 10.5271/sjweh.3833 31134284

[6] EU-OSHA. Motivation for Employers to Carry Out Workplace Health Promotion. Luxembourg: Publications Office of the European Union (2012).

[7] Wierenga D Engbers L Van Empelen P Duijts S Hildebrandt V Van Mechelen W. What is actually measured in process evaluations for worksite health promotion programs: a systematic review. BMC Public Health. (2013) 13:1190. 10.1186/1471-2458-13-1190 24341605 PMC3890539

[8] Glasgow R Lichtenstein E Marcus A. Why don't we see more translation of health promotion research to practice? Rethinking the efficacy-to-effectiveness transition. Am J Public Health. (2003) 93:1261–7. 10.2105/AJPH.93.8.1261 12893608 PMC1447950

[9] Crump CE Earp JA Kozma CM Hertz-Picciotto I. Effect of organization-level variables on differential employee participation in 10 federal worksite health promotion programs. Health Educ Q. (1996) 23:204–23. 10.1177/109019819602300206 8744873

[10] Robroek SJW van Lenthe FJ van Empelen P Burdorf A. Determinants of participation in worksite health promotion programmes: a systematic review. Int J Behav Nutr Phys Act. (2009) 6:26. 10.1186/1479-5868-6-26 19457246 PMC2698926

[11] Bull SS Gillette C Glasgow RE Estabrooks P. Work site health promotion research: to what extent can we generalize the results and what is needed to translate research to practice? Health Educ Behav. (2003) 30:537–49. 10.1177/1090198103254340 14582596

[12] Goetzel R Henke RM Tabrizi M Pelletier K Loeppke R Ballard D. Do workplace health promotion (wellness) programs work? J Occup Environ Med. (2014) 56:927–34. 10.1097/JOM.0000000000000276 25153303

[13] Wolfenden L Goldman S Stacey F Grady A Kingsland M Williams C. Strategies to improve the implementation of workplace-based policies or practices targeting tobacco, alcohol, diet, physical activity and obesity. Cochrane Database Syst Rev. (2018) 11:CD012439. 10.1002/14651858.CD012439.pub2 30480770 PMC6362433

[14] Raaijmakers T Van Dijk S. Gezondheidsbevordering op de Werkplek. Ondersteuningsbehoefte van professionals werkzaam in de publieke setting. RIVM briefrapport. (2012).

[15] CHRODIS. Workplace Health Promotion: Lombardy WHP Network Italy. Lombardy (2017).

[16] CHRODIS. Joint Action on Chronic Diseases & Promoting Healthy Ageing across the Life Cycle - Good Practices in Health Promotion & Primary Prevention of Chronic Diseases. Summary Report. (2014).

[17] Nyumba T. Wilson, Derrick C, Mukherjee N. The use of focus group discussion methodology: insights from two decades of application in conservation. Methods Ecol Evol. (2018) 9:20–32. 10.1111/2041-210X.12860

[18] United Nations. Statistical D. International Standard industrial classification of all economic activities (ISIC): Revised 4. New York, NY: United Nations (2008).

[19] Green J Thorogood N. Qualitative Methods for Health Research. Thousand Oaks, CA: SAGE Publications (2018).

[20] Damschroder LJ Lowery JC. Evaluation of a large-scale weight management program using the consolidated framework for implementation research (CFIR). Implement Sci. (2013) 8:51. 10.1186/1748-5908-8-51 23663819 PMC3656778

[21] Kirk MA Kelley C Yankey N Birken SA Abadie B Damschroder L. Systematic review of the use of the consolidated framework for implementation research. Implement Sci. (2016) 11:72. 10.1186/s13012-016-0437-z 27189233 PMC4869309

[22] What is the CFIR? (2021). Available online at: https://cfirguide.org/ (accessed April 7, 2021).

[23] Braun V Clarke V. Using thematic analysis in psychology. Qual Res Psychol. (2006) 3:77–101. 10.1191/1478088706qp063oa

[24] Fereday J Muir-Cochrane E. Demonstrating rigor using thematic analysis: a hybrid approach of inductive and deductive coding and theme development. Int J Qual Methods. (2006) 5:80–92. 10.1177/160940690600500107

[25] Moser A Korstjens I. Series: practical guidance to qualitative research. Part 3: sampling, data collection and analysis. Eur J Gen Prac. (2017) 24:9–18. 10.1080/13814788.2017.1375091 29199486 PMC5774281

[26] Birken BE Linnan LA. Implementation challenges in worksite health promotion programs. N C Med J. (2006) 67:438. 10.18043/ncm.67.6.438 17393707

[27] Biswas A Begum M Van Eerd D Johnston H Smith PM Gignac MAM. Integrating safety and health promotion in workplaces: a scoping review of facilitators, barriers, and recommendations. Health Promot Pract. (2022) 23:984–98. 10.1177/15248399211028154 34596446

[28] Christensen JR Larsen CM Kolind MI. Managers attitude towards implementing workplace health promotion programmes to employees in eldercare: a cross-sectional study. Public Health Pract. (2020) 1:100049. 10.1016/j.puhip.2020.100049 36101701 PMC9461371

[29] Eriksson A editor. Health-Promoting Leadership: A Study of the Concept and Critical Conditions for Implementation and Evaluation [Ph.D. dissertation]. Gothenburg, IL: Nordic School of Public Health (2011).

[30] Ruiz-Dominguez F Stegeman I Dolz-López J Papartyte L Fernández-Pérez D. Transfer and implementation process of a good practice in workplace health promotion. Int J Environ Res Public Health. (2021) 18:5254. 10.3390/ijerph18105254 34069229 PMC8155958

[31] Pescud M Teal R Shilton T Slevin T Ledger M Waterworth P. Employers' views on the promotion of workplace health and wellbeing: a qualitative study. BMC Public Health. (2015) 15:642. 10.1186/s12889-015-2029-2 26162910 PMC4499225

[32] Crane M Bohn-Goldbaum E Lloyd B Rissel C Bauman A Indig D. Evaluation of Get Healthy at Work, a state-wide workplace health promotion program in Australia. BMC Public Health. (2019) 19:183. 10.1186/s12889-019-6493-y 30760237 PMC6373144

[33] Rojatz D Merchant A Nitsch M. Factors influencing workplace health promotion intervention: a qualitative systematic review. Health Promot Int. (2017) 32:831–9. 10.1093/heapro/daw015 27006365

[34] Linnan L Bowling M Childress J Lindsay G Blakey C Pronk S. Results of the 2004 national worksite health promotion survey. Am J Public Health. (2008) 98:1503–9. 10.2105/AJPH.2006.100313 18048790 PMC2446449

[35] NIOSH. Essential Elements of Effective Workplace Programs. CDC (2015). Available online at: https://www.cdc.gov/niosh/TWH/essentials.html (accessed March 25 , 2022).

[36] Collier D Mahoney J. Insights and pitfalls: selection bias in qualitative research. World Polit. (1996) 49:56–91. 10.1353/wp.1996.002319051285

[37] O'Donnell A Cummins D. The use of qualitative methods to research networking in SMEs. Qual Mark Res. (1999) 2:82–91. 10.1108/13522759910269991

[38] Dos Santos Marques IC Theiss LM Johnson CY McLin E Ruf BA Vickers SM. Implementation of virtual focus groups for qualitative data collection in a global pandemic. Am J Surg. (2021) 221:918–22. 10.1016/j.amjsurg.2020.10.009 33070983 PMC7550163

[39] Tates K Zwaanswijk M Otten R van Dulmen S Hoogerbrugge PM Kamps WA. Online focus groups as a tool to collect data in hard-to-include populations: examples from paediatric oncology. BMC Med Res Methodol. (2009) 9:15. 10.1186/1471-2288-9-15 19257883 PMC2653071

[40] Kite J Phongsavan P. Insights for conducting real-time focus groups online using a web conferencing service. F1000Res. (2017) 6:122. 10.12688/f1000research.10427.2 28781752 PMC5527981

[41] Janghorban R Latifnejad Roudsari R Taghipour A. Skype interviewing: the new generation of online synchronous interview in qualitative research. Int J Qual Stud Health Well-being. (2014) 9:24152. 10.3402/qhw.v9.24152 24746247 PMC3991833

[42] Damschroder L Aron D Keith R Kirsh S Alexander J Lowery J. Fostering implementation of health services research findings into practice: a consolidated framework for advancing implementation science. Implement Sci. (2009) 4:50. 10.1186/1748-5908-4-50 19664226 PMC2736161

[43] Robroek S Vathorst S Hilhorst M Burdorf A. Moral issues in workplace health promotion. Int Arch Occup Environ Health. (2011) 85:327–31. 10.1007/s00420-011-0675-y 21710278 PMC3299975

[44] Strickland JR Kinghorn AM Evanoff BA Dale AM. Implementation of the healthy workplace participatory program in a retail setting: a feasibility study and framework for evaluation. Int J Environ Res Public Health. (2019) 16:590. 10.3390/ijerph16040590 30781669 PMC6406806

[45] van der Feltz S van der Molen HF Lelie L Hulshof CTJ van der Beek AJ Proper KI. Changes in fruit and vegetable consumption and leisure time physical exercise after a citizen science-based worksite health promotion program for blue-collar workers. Int J Environ Res Public Health. (2022) 19:13652. 10.3390/ijerph192013652 36294231 PMC9603698

[46] CFIR. Qualitative Data 2021. Available online at: https://cfirguide.org/evaluation-design/qualitative-data/ (accessed December 7, 2021).

